# Lens differentiation is controlled by the balance between PDGF and FGF signaling

**DOI:** 10.1371/journal.pbio.3000133

**Published:** 2019-02-04

**Authors:** Hongge Li, Yingyu Mao, Michael Bouaziz, Honglian Yu, Xiuxia Qu, Fen Wang, Gen-Sheng Feng, Carrie Shawber, Xin Zhang

**Affiliations:** 1 Departments of Ophthalmology, Pathology and Cell Biology, Columbia University, New York, New York, United States of America; 2 Department of Biochemistry, School of Basic Medicine, Jining Medical University, Jining, Shandong, China; 3 Wuxi School of Medicine, Jiangnan University, Wuxi, Zhejiang China; 4 Center for Cancer Biology and Nutrition, Texas A&M University, Houston, Texas, United States of America; 5 Department of Pathology, University of California San Diego, La Jolla, California, United States of America; 6 Department of Obstetrics and Gynecology, Columbia University, New York, New York, United States of America; California Institute of Technology, UNITED STATES

## Abstract

How multiple receptor tyrosine kinases coordinate cell fate determination is yet to be elucidated. We show here that the receptor for platelet-derived growth factor (PDGF) signaling recruits the p85 subunit of Phosphoinositide 3-kinase (PI3K) to regulate mammalian lens development. Activation of PI3K signaling not only prevents B-cell lymphoma 2 (BCL2)-Associated X (Bax)- and BCL2 Antagonist/Killer (Bak)-mediated apoptosis but also promotes Notch signaling to prevent premature cell differentiation. Reducing PI3K activity destabilizes the Notch intracellular domain, while the constitutive activation of Notch reverses the PI3K deficiency phenotype. In contrast, fibroblast growth factor receptors (FGFRs) recruit Fibroblast Growth Factor Receptor Substrate 2 (Frs2) and Rous sarcoma oncogene (Src) Homology Phosphatase 2 (Shp2) to activate Mitogen-Activated Protein Kinase (MAPK) signaling, which induces the Notch ligand Jagged 1 (Jag1) and promotes cell differentiation. Inactivation of Shp2 restored the proper timing of differentiation in the *p85* mutant lens, demonstrating the antagonistic interaction between FGF-induced MAPK and PDGF-induced PI3K signaling. By selective activation of PI3K and MAPK, PDGF and FGF cooperate with and oppose each other to balance progenitor cell maintenance and differentiation.

## Author summary

A central aim in understanding cell signaling is to decode the cellular logic that underlies the functional specificity of growth factors. Although these factors are known to activate a common set of intracellular pathways, they nevertheless play specific roles in development and physiology. Using lens development in mice as a model, we show that fibroblast growth factor (FGF) and platelet-derived growth factor (PDGF) antagonize each other through their intrinsic biases toward distinct downstream targets. While FGF primarily induces the Ras–Mitogen-Activated Protein Kinase (MAPK) axis to promote lens cell differentiation, PDGF preferentially stimulates Phosphoinositide 3-kinase (PI3K) to enhance Notch signaling, which is necessary for maintaining the lens progenitor cell pool. By revealing the intricate interactions between PDGF, FGF, and Notch, we present a paradigm for how signaling crosstalk enables balanced growth and differentiation in multicellular organisms.

## Introduction

**Fig 1:**
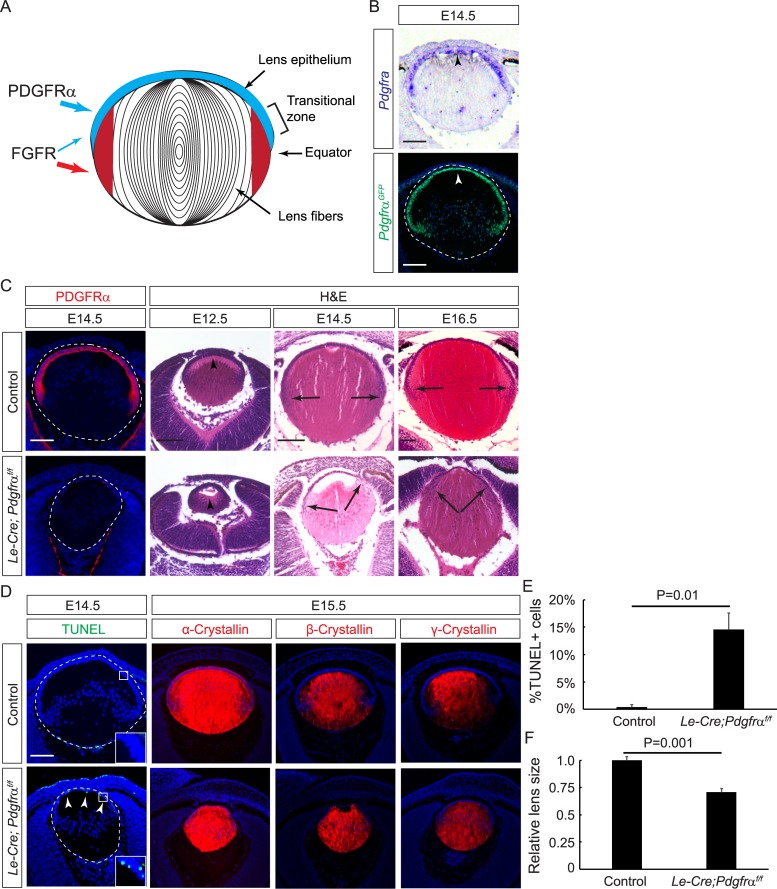
PDGFRα is essential for maintaining the lens epithelial cell population. **(A)** Schematic diagram of the mammalian lens. PDGFRα is expressed in the lens epithelial cells (blue), whereas FGFRs are predominantly expressed in the newly differentiated lens fiber cells (red). **(B)** In situ hybridization and immunofluorescence staining showed that *Pdgfrα* was expressed exclusively in the anterior epithelium of the E14.5 lens (arrowheads). **(C)** The *Pdgfrα* KO lens lost PDGFRα immunostaining by E14.5. The elongation of primary lens fiber cells was retarded at E12.5 (arrowhead), and the transitional zone was shifted anteriorly at E14.5 and E16.5 (arrows). **(D)** The *Pdgfrα* mutant lens displayed aberrant levels of apoptosis as indicated by TUNEL staining, while the expression of crystallins was unaffected. **(E)** Quantitation of TUNEL-positive cells as the percentage of total number of lens epithelial cells marked by DAPI at E14.5. Student *t* test, *P* = 0.01, *n* = 3 embryos. (**F**) Quantification of the relative lens size at E14.5. Student *t* test, *P* = 0.001, *n* = 4 embryos. Scale bars: 100 μm. The numerical data used in panels E and F are included in S1 Data. E, embryonic day; FGF, fibroblast growth factor; FGFR, FGF receptor; HE, hematoxylin–eosin; KO, knockout; PDGFRα, platelet-derived growth factor receptor α.

Receptor Tyrosine Kinases (RTKs) are a large family of membrane proteins that can activate a common set of downstream pathways, but they are also known to elicit distinct biological responses. This raises the question of how the signaling specificities of these receptors are generated. The vertebrate lens is a unique model to study the functional mechanism of RTKs. It is composed of an epithelial monolayer overlying a lens-fiber–cell core that is devoid of the complications encountered with vasculature invasion, neural innervation, and immune infiltration [1, 2]. During embryonic development, lens progenitor cells within the epithelium proliferate and migrate toward the equator of the lens until they reach the transitional zone, where they exit the cell cycle and begin to differentiate into lens fiber cells (Fig 1A). Previous studies have identified several RTKs in the lens. Among them, fibroblast growth factor receptors (FGFRs) are expressed weakly in the lens epithelium but strongly in the elongating secondary fiber cells present in the equator region [3]. Indeed, in lens explant cultures, FGFs have been shown to promote either epithelial cell proliferation or fiber-cell differentiation in a dose-dependent manner [4]. This is supported by in vivo evidence that transgenic expressions of FGFs cause premature differentiation of lens epithelial cells into fiber cells, while deletion of FGFRs or their coreceptor heparan sulfates abrogate lens fiber differentiation [5–8].

Another RTK known as platelet-derived growth factor receptor α (PDGFRα) is restricted to the lens epithelium (Fig 1A). Its ligands PDGFA and PDGFB are present in the ciliary margin zone, which abuts the transitional zone of the lens epithelium [9, 10]. PDGF in explant cultures was reported to promote the proliferation of lens epithelial cells and potentiates FGF-induced differentiation [10–12]. In support of this, transgenic overexpression of *PDGFA* not only increases lens size but also induces a slight elongation of lens epithelium cells, which is characteristic of lens-fiber–cell differentiation [9]. Moreover, an early examination of the *Patch* mutant mouse, which harbors a large genomic deletion encompassing the *Pdgfrα* locus, reported lens fiber defects [13]. However, this claim was later contradicted by the targeted deletion of the *Pdgfrα* gene, which did not appear to affect lens development. Nonetheless, it should be noted that a detailed analysis of the *Pdgfrα* null lens was never described [14, 15]. Therefore, the role of PDGF signaling in lens development remains a topic requiring further exploration.

In this study, we investigated PDGF signaling in lens development, aiming to understand its crosstalk with the closely related FGF-signaling pathway. We showed that PDGF signaling primarily activates the Phosphoinositide 3-kinase (PI3K)–Protein kinase B (AKT) pathway in lens development and the direct binding of PI3K to PDGFRα is required for preventing the depletion of lens epithelial cells. In contrast, FGF and Mitogen-Activated Protein Kinase (MAPK) signaling display the opposite effect in promoting lens-fiber–cell differentiation. Attenuation of MAPK signaling restored the proper balance of lens progenitor and differentiated cells in PI3K mutants, demonstrating their antagonistic roles in lens development. These two intracellular pathways converge upon Notch signaling because MAPK is required for the expression of Jagged 1 (Jag1) and PI3K is necessary for the stability of the Notch intracellular domain (NICD). Taken together, PDGF–PI3K signaling counterbalances FGF–MAPK to maintain the progenitor pool of cells within the lens epithelium.

## Results

### The PDGFR–PI3K signaling axis regulates lens development

At mouse embryonic day 14.5 (E14.5), *Pdgfrα* is exclusively expressed in the lens epithelium, as indicated by RNA in situ hybridization and a green fluorescent protein (GFP) reporter from the *Pdgfrα* locus (Fig 1B, arrowheads). We ablated *Pdgfrα* using the Cre deleter *Le-Cre*, which is active in the lens precursor cells as early as E9.5 [16]. As expected, this led to the complete loss of PDGFRα immunostaining in the *Le-Cre;Pdgfrα*^*f/f*^ lens (Fig 1C, circled in dotted lines). At E12.5, the primary fiber cells in the *Le-Cre;Pdgfrα*^*f/f*^ mutant failed to reach the anterior rim of the lens as those in the control samples (*Le-Cre* or *Le-Cre; Pdgfrα* ^*f/+*^) did (Fig 1C, arrowheads). At E14.5 and E16.5, lens epithelial cells initiated differentiation into the secondary fiber cells at the transitional zone, which was located at the equator region of the control lens. In *Pdgfrα* mutants, the transitional zone had shifted anteriorly, resulting in a significant shortening of the lens epithelium (Fig 1C, arrows). Furthermore, there was an abnormal increase in cell death, as shown by TUNEL staining in the lens epithelium and reduction in lens size, while the expression of α-, β-, and γ-crystallins in lens fiber cells was preserved (Fig 1D–1F, arrowheads). These results showed that PDGF signaling was required to maintain the balance between lens epithelial and fiber-cell compartments.

**Fig 2:**
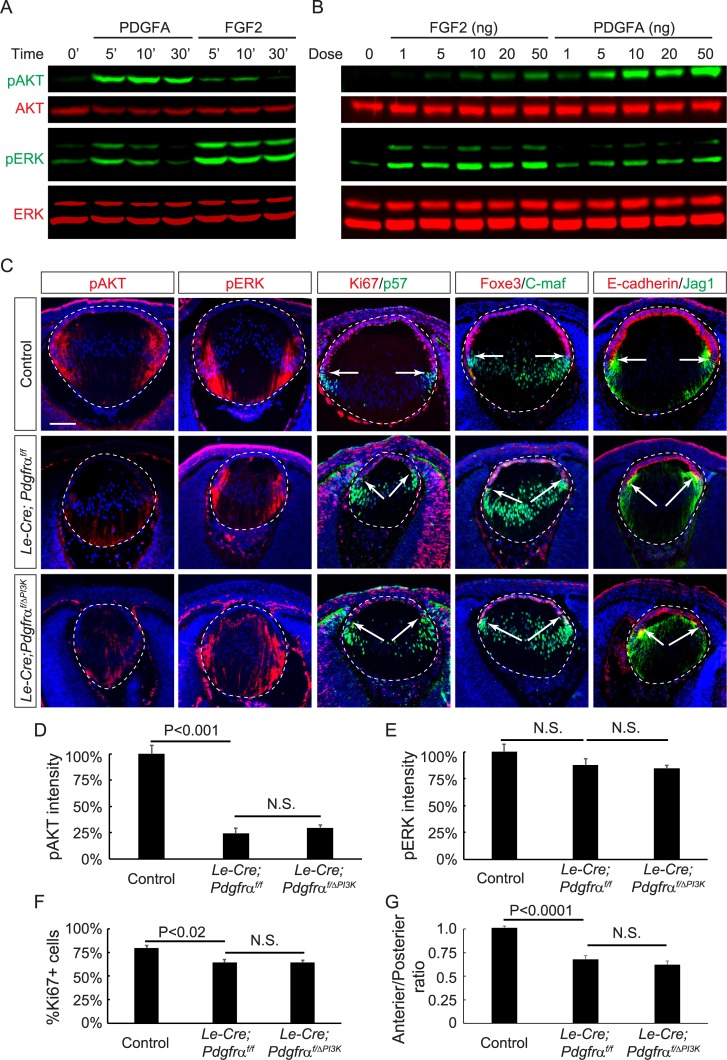
PI3K–AKT signaling is regulated by PDGFRα during lens development. **(A–B)** AKT phosphorylation was strongly stimulated by PDGFA but weakly by FGF2 in immortalized lens cells. In contrast, FGF2, but not PDGFA, induced strong ERK phosphorylation. (**C**) pAKT was down-regulated in mutant lenses lacking *Pdgfrα* (*Le-Cre;Pdgfrα*^*f/f*^) and in lenses expressing a mutant allele of *Pdgfra* that prevents interaction with PI3K (*Le-Cre; Pdgfrα*^*f/ΔPI3K*^). However, ERK phosphorylation was unaffected in either *Pdgfra* mutant. Staining of the anterior progenitor markers Ki67, Foxe3, and E-cadherin and posterior differentiation markers p57, C-maf, and Jag1 indicated the anterior shift of the transitional zone in these mutants (arrows). (**D–E**) The relative fluorescent intensities of pAKT and pERK at the transitional zones of the lenses. One-way ANOVA test, *n* = 3 embryos for each genotype. (**F**) The percentage of Ki67+ cells in the lens epithelium. One-way ANOVA test, *n* = 3 embryos for each genotype. (**G**) Quantification of the lens perimeter covering the anterior epithelium versus that of the posterior lens fiber. One-way ANOVA test, *n* = 5 embryos for *Le-Cre;Pdgfrα*^*f/f*^ and control, *n* = 6 for *Le-Cre; Pdgfrα*^*f/ΔPI3K*^. Scale bar: 100 μm. The numerical data used in panels D, E, F, and G are included in S1 Data. AKT, Protein kinase B; C-maf, MAF BZIP Transcription Factor; ERK, Extracellular Signal-Regulated Kinase; FGF2, fibroblast growth factor 2; Foxe3, Forkhead Box E3; Jag1, Jagged 1; N.S., not significant; pAKT, phospho-AKT; PDGFRα, Platelet-Derived Growth Factor Receptor α; pERK, phospho-ERK; PI3K, Phosphoinositide 3-kinase.

Similar to other RTK family members, PDGF and FGF signaling can induce a common set of downstream effectors, notably Ras–MAPK and PI3K–AKT [17, 18]. To determine which intracellular pathway mediates the effect of PDGF signaling in the lens, we generated immortalized lens epithelial cells from neonatal pups [19], which expressed a mixture of progenitor markers Paired Box 6 (Pax6) and α-crystallin and more differentiated markers β- and γ-crystallin (S1 Fig). As shown in Fig 2A, FGF2 at 50 ng/ml induced a strong elevation in Extracellular Signal-Regulated Kinase (ERK) phosphorylation that lasted for 30 minutes, but the increase in phospho-AKT (pAKT) was much weaker and transient. Conversely, the same concentration of PDGFA produced a much higher phosphorylation rate of AKT than that of ERK. From 1 ng/ml to 50 ng/ml, PDGFA consistently stimulated higher pAKT levels than FGF2 at 5 minutes, whereas FGF2 generated stronger phospho-ERK (pERK) response than PDGFA (Fig 2B). These results were consistent with previous observations that FGF preferentially activated Ras–MAPK signaling, while PDGF was more biased toward PI3K–AKT signaling [20–24]. Indeed, pAKT staining was significantly reduced in the *Le-Cre;Pdgfrα*^*f/f*^ mutant lens, with pERK being maintained at normal levels (Fig 2C–2E). To confirm that AKT phosphorylation in the lens depended on PDGFRα-stimulated PI3K signaling, we took advantage of the *Pdgfrα*^*ΔPI3K*^ knock-in mutant that lacks the docking site for PI3K [20]. In *Le-Cre; Pdgfrα*^*f/ΔPI3K*^ lenses, loss of the PDGFRα–PI3K interaction failed to disrupt pERK staining in the transitional zone, with the levels of pAKT being comparably reduced as in the *Le-Cre;Pdgfrα*^*f/f*^ lens (Fig 2B–2E). Moreover, both mutants displayed the anterior encroachment of p57 staining, which marked the differentiating lens cells that had just exited the cell cycle (Fig 2B, arrows). There were fewer numbers of proliferative Ki67-expressing cells (Fig 2F), indicating a depletion of the lens progenitor cell pool. This was further demonstrated by the staining of epithelial cell markers Forkhead Box E3 (Foxe3) and E-cadherin and fiber-cell markers MAF BZIP Transcription Factor (C-maf) and Jag1, which together displayed a significant reduction in the length of the anterior lens epithelium in relation to the posterior lens circumference (Fig 2G). These observations were consistent with the notion that PDGFR–PI3K signaling maintains the progenitor population of the lens epithelium.

### Ablation of PI3K phenocopies *Pdgfrα* mutant lens defects

**Fig 3:**
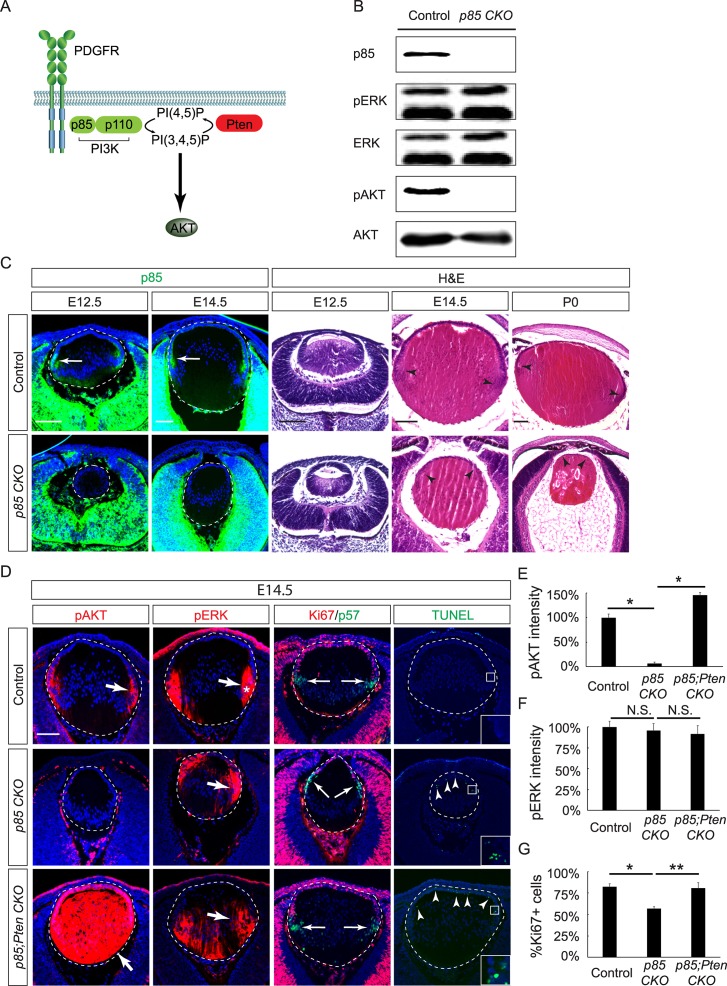
Conditional KO of the PI3K subunit p85 phenocopies the *Pdgfrα* mutant lens. **(A)** The PDGFR recruits the p85 subunit of PI3K, which activates AKT signaling by converting PI-4,5-P to PI-3,4,5-P. This reaction can be reserved by Pten to inhibit AKT signaling. **(B)** Western blot analysis showed the loss of p85 and pAKT, but not pERK, in E16.5 *p85 CKO* lens lysates. **(C)** p85 immunostaining was primarily localized to the transitional zone of the control lens (arrows) but lost in *p85 CKO* mutants. HE staining revealed the retarded elongation of primary fiber cells at E12.5 and the anterior shift of the transitional zones at E14.5 and P0 (arrowheads). **(D)** The *p85 CKO* lens showed normal pERK staining but lost pAKT in the transitional zone (arrows). The anterior lens epithelium displayed extensive TUNEL staining, indicative of apoptosis, and the encroachment of p57 positive cells that suggests the premature differentiation of progenitor cells. Further deletion of *Pten* in *p85 CKO* led to the up-regulation of pAKT in the entire lens but a modest increase in pERK. Although the apoptosis defect was not rescued by *Pten* deletion, the transitional zone in *p85;Pten CKO* lens marked by p57 staining was reverted back to the equatorial region of the lens. (**E–F**) The relative fluorescent intensities of pAKT and pERK at the transitional zones of the lenses. One-way ANOVA test, *P < 0.0001, *n* = 3 embryos for each genotype. (**G**) The percentage of Ki67+ cells in the lens epithelium. One-way ANOVA test, *P < 0.01, **P < 0.02, *n* = 3 embryos for each genotype. The numerical data used in panels E, F, and G are included in S1 Data. AKT, Protein kinase B; E, embryonic day; ERK, Extracellular Signal-Regulated Kinase; HE, hematoxylin–eosin; KO, knockout; pAKT, phospho-AKT; PDGFR, platelet-derived growth factor receptor; pERK, phospho-ERK; PI3K, Phosphoinositide 3-kinase; PI-4,5-P, Phosphatidylinositol 4,5-bisphosphate; Pten, phosphatase and tensin homolog; *p85 CKO*, *Le-Cre;p85α*^*f/f*^*;p85β*^*KO/KO*^.

**Fig 4:**
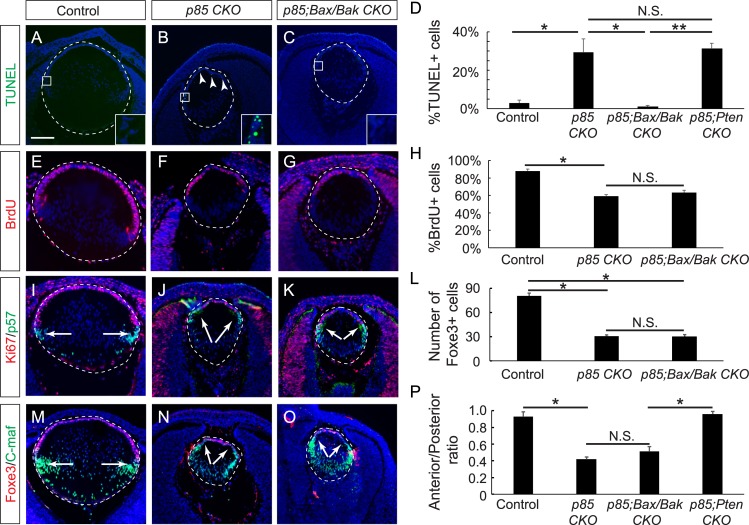
*Bax/Bak* mutations rescue cell apoptosis but not the loss of lens progenitor cells in *p85 CKO*. **(A–D)** *Bax/Bak* deletion completely blocked the abnormal cell death observed in the *p85 CKO* lens, as shown by TUNEL staining (one-way ANOVA test, *P < 0.02, **P < 0.01, *n* = 4 embryos for *p85 CKO*, *n* = 3 embryos for other genotypes). **(E–H)** The cell proliferation as indicated by the incorporation of BrdU was significantly reduced in both *p85 CKO* and *p85;Bax/Bak CKO* (one-way ANOVA test, *P < 0.0001, *n* = 3 embryos for each genotype). **(I–O)** Ki67, p57, Foxe3, and C-maf immunostaining showed that the deletion of *Bax* and *Bak* did not revert the anterior shift of the transitional zone (one-way ANOVA test, *P < 0.0001, *n* = 3 embryos for each genotype). **(O)** The length of the anterior epithelium was quantified against the perimeter of the posterior lens-fiber–cell compartment. One-way ANOVA test, *P < 0.0001, *n* = 9 embryos for the control, *n* = 7 for *p85 CKO*, *n* = 10 for *p85;Bax/Bak CKO*, *n* = 5 embryos for *p85;Pten CKO*. Scale bar: 100 μm. The numerical data used in panels D, H, L, and P are included in S1 Data. Bak, BCL2 Antagonist/Killer; Bax, BCL2-Associated X; BCL2, B-cell lymphoma 2; BrdU, Bromodeoxyuridine; C-maf, MAF BZIP Transcription Factor; Foxe3, Forkhead Box E3; KO, knockout; N.S., not significant; Pten, phosphatase and tensin homolog; *p85;Bax/Bak CKO*, *Le-Cre;p85α*^*f/f*^*;p85β*^*KO/KO*^*Bax*^*flox/flox*^*;Bak*^*KO/KO*^; *p85 CKO*, *Le-Cre;p85α*^*f/f*^*;p85β*^*KO/KO*^; *p85;Pten*, *Le-Cre;p85α*^*f/f*^*;p85β*^*KO/KO*^*;Pten*^*f/f*^.

PI 3-kinase is a heterodimer composed of two subunits: a regulatory one known as p85 and a catalytic one known as p110. When PI3K binds to the specific phosphotyrosine residues of RTKs, p85 brings p110 to the plasma membrane to catalyze the conversion of Phosphatidylinositol 4,5-bisphosphate (PI-4,5-P) to Phosphatidylinositol (3,4,5)-trisphosphate (PI-3,4,5-P) (Fig 3A). We thus sought out to abolish PI3K signaling in the lens by crossing *Le-Cre* with the floxed allele of *Pik3r1* encoding p85α/p55α/p50α and a knockout (KO) allele of *Pik3r2* encoding p85β [25, 26]. Western blot analysis demonstrated that *Le-Cre;p85α* ^*f/f*^*;p85β* ^*KO/KO*^ (*p85 CKO*) mutant lenses lost both p85 expression and AKT phosphorylation, but the pERK level was unchanged, consistent with the role of PI3K in AKT activation (Fig 3B). In line with a previous report, immunostaining showed that p85 was predominantly expressed in the transitional zone of the control lens, overlapping with pAKT staining (Fig 3C, arrows) [27]. In *p85 CKO* mutants, loss of p85 resulted in a smaller lens than the controls as early as E12.5 (Fig 3C). Histology analysis showed that the transitional zone in the *p85 CKO* lens also moved anteriorly (Fig 3C, arrowheads, quantified in Fig 4P). Altogether, the *p85 CKO* closely resembled the *Pdgfrα* mutant in their lens phenotypes, further demonstrating that PI3K is the main effector of PDGF signaling in the lens.

Knowing that phosphatase and tensin homolog (Pten) reverses the lipid phosphorylation reaction catalyzed by PI3K, we reasoned that the removal of *Pten* may ameliorate the *p85 CKO* mutant lens phenotype. Indeed, *Le-Cre;p85α* ^*f/f*^*;p85β* ^*KO/KO*^*;Pten* ^*f/f*^ (*p85;Pten CKO*) mutants displayed hyperphosphorylation of AKT in the entire lens, whereas pERK staining was only modestly expanded (Fig 3D–3F, arrows). Marked by the boundary between Ki67 and p57 staining, the transitional zone moved anteriorly in *p85 CKO* mutants, but it reverted back to the lens equator in *p85;Pten CKO* mutants (Fig 3D, arrows). Similar to *Pdgfrα* mutants, the *p85 CKO* lens exhibited aberrant cell death in the anterior epithelium, as indicated by TUNEL staining (Fig 3D, arrowheads), and reduction in cell proliferation, as shown by Ki67 expression (Fig 3G). It was previously reported that deletion of *Pten* alone in the lens led to slight increase in cell proliferation and apoptosis at E12.5 [28]. The loss of *Pten* in *p85;Pten CKO* mutants restored cell proliferation, but it did not prevent cell apoptosis defect. This result indicated that *Pten* deletion rescued the *p85* deficiency in the epithelial and fiber-cell compartmentation without enhancing cell survival.

### PI3K maintains the lens progenitor pool independently of its role in cell survival

The depletion of the lens progenitor pool in *Pdgfrα* and *p85* mutants could be due to the increase in cell death present in the lens epithelium. However, this model was challenged by the selective rescue of the lens epithelial population but not cell survival in *p85;Pten CKO* mutants. To address the question of whether cell death was responsible for depleting the lens progenitors, we sought to disable the intrinsic apoptotic pathway in *p85* mutants. The proapoptotic B-cell lymphoma 2 (BCL2) proteins BCL2-Associated X (Bax) and BCL2 Antagonist/Killer (Bak) are required for permeabilization of the mitochondrial outer membrane and release of cytochrome c into the cytoplasm, which triggers the programmed cell death pathway [29, 30]. Previous studies have shown that knocking out Bax and Bak prevents most forms of apoptosis in vivo [31, 32]. After crossing *p85 CKO* mutants with *Bax*^*flox/flox*^*;Bak*^*KO/KO*^ mice, we confirmed by TUNEL staining that genetic removal of Bax and Bak indeed prevented cell apoptosis in *Le-Cre;p85α* ^*f/f*^*;p85β* ^*KO/KO*^*;Bax*^*flox/flox*^*;Bak*^*KO/KO*^ (*p85;Bax/Bak CKO*) mutant lenses (Fig 4A–4D, arrowheads), but the cell proliferation defect was not rescued, as shown by the Bromodeoxyuridine (BrdU) incorporation index (Fig 4E–4H). Moreover, Ki67 and p57 staining showed that the transitional zone was still positioned anteriorly in the *p85;Bax/Bak CKO* mutant lens (Fig 4I–4K, arrows). Further, the number of lens epithelial cells marked by Foxe3 staining remained significantly reduced compared to the control lens (Fig 4L–4O, arrows). As a result, the ratios of the anterior epithelium versus the posterior lens circumference were indistinguishable between *p85 CKO* and *p85;Bax/Bak CKO* mutant lenses (Fig 4P). Taken together, these results demonstrated that the cell death caused by PI3K inactivation was not a key factor in the depletion of lens progenitors.

### PI3K is required for Notch signaling in lens epithelial cells

**Fig 5:**
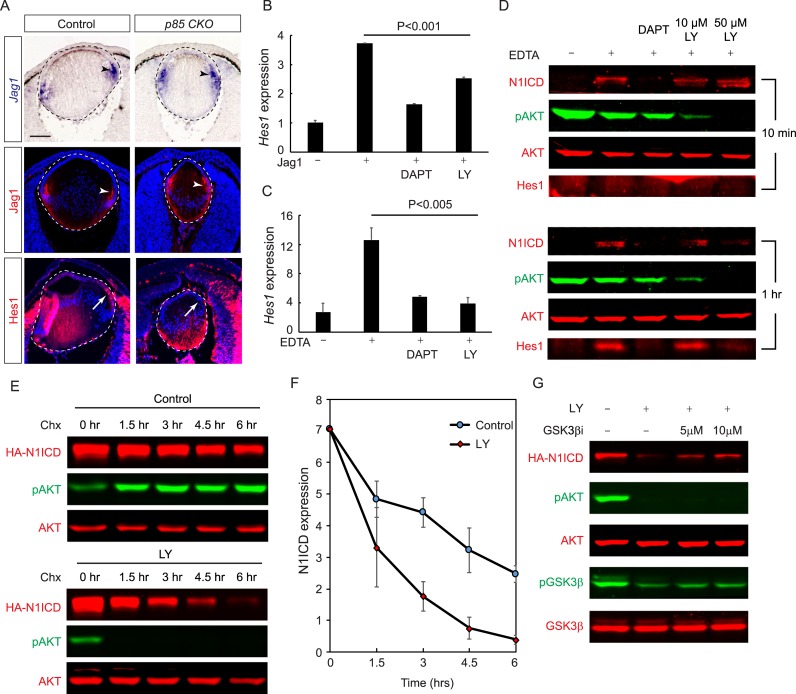
Loss of PI3K activity reduces Notch signaling by decreasing N1ICD stability. **(A)** The *p85 CKO* lens displayed normal expression levels of Jag1 in the nascent lens fiber cells (arrowheads) but a sharp reduction of Hes1 in the lens progenitor cells (arrows). **(B)** Both the Notch signaling inhibitor DAPT and the PI3K inhibitor LY294002 reduced Jag1-induced *Hes1* expression in immortalized lens cells. **(C)** Hes1 expression induced by EDTA in lens cell culture was also suppressed by DAPT and LY294002. **(D)** EDTA induced N1ICD in lens cells at 10 minutes and Hes1 by 1 hour, and both were inhibited by DAPT. In contrast, increasing concentration of LY294002 suppressed AKT phosphorylation, and it affected N1ICD level after 1 hour. **(E)** After treatment of Chx to stop protein translation in lens cells, there was a slow decay of exogenously expressed HA-tagged rat-N1ICD, which was greatly accelerated in the presence of 50 μM LY294002. **(F)** Quantification of the HA-N1ICD level normalized with AKT expression after three independent experiments. **(G)** GSK3βi CHIR99021 partially stabilized HA-N1ICD after LY294002 treatment. The numerical data used in panels B, C, and F are included in S1 Data. AKT, Protein kinase B; Chx, cycloheximide; DAPT, N-[N-(3,5-Difluorophenacetyl)-L-alanyl]-S-phenylglycine t-butyl ester; GSK3β, Glycogen Synthase Kinase 3β; GSK3βi, GSK3β inhibitor; HA, Human influenza hemagglutinin; Hes1, Hes Family BHLH Transcription Factor 1; Jag1, Jagged 1; KO, knockout; N1ICD, Notch1 intracellular domain; pAKT, phsopho-AKT; PI3K, Phosphoinositide 3-kinase; pGSK3β, phospho-GSK3β; *p85 CKO*, *Le-Cre;p85α*^*f/f*^*;p85β*^*KO/KO*^.

The loss of lens epithelial progenitors observed in *Pdgfra* and *p85* mutants was highly reminiscent of the defects observed in the Notch-signaling mutant lens [33–37]. During lens development, the Notch ligand Jag1 is expressed by the nascent secondary fiber cells at the equatorial region of the lens. It induces Notch signaling to activate Hes Family basic helix–loop–helix (BHLH) Transcription Factor 1 (Hes1) in the transitional zone, promoting the proliferation of the lens epithelial cells and preventing them from premature differentiation. Genetic deletion of Jag1 resulted in a striking loss of the anterior epithelial cells [34]. Although *p85 CKO* mutants displayed a similar depletion of lens epithelial cells, Jag1 mRNA and protein expression were preserved (Fig 5A, arrowheads). Instead, there was a striking loss of Hes1 expression in the transitional zone (Fig 5A, arrow), suggesting that PI3K may be required for Notch-signaling recipient cells in the lens epithelium.

To investigate the molecular mechanism of PI3K in regulating Notch signaling, we cultured the immortalized lens epithelial cells on plates coated with Jag1 and found that these cells displayed elevated levels of *Hes1* expression as measured by quantitative Reverse transcription-polymerase chain reaction (RT-PCR) (Fig 5B). Importantly, the Jag1-induced *Hes1* expression was repressed by both the Notch inhibitor N-[N-(3,5-Difluorophenacetyl)-L-alanyl]-S-phenylglycine t-butyl ester (DAPT) and the PI3K inhibitor LY294002, which is consistent with the in vivo observation that inactivation of PI3K disrupted Jag1-induced Notch signaling in the lens. We next sought to bypass Jag1 in vitro by using EDTA to chelate the extracellular calcium, which stimulates shedding of the Notch extracellular domain and release of the NICD to activate *Hes1* transcription [38, 39]. In the lens cell cultures, the EDTA-induced *Hes1* expression was also suppressed by DAPT and LY294002 (Fig 5C). Taken together, these results suggest that PI3K may regulate the Notch signaling pathway downstream of the Jag1 ligand activation of Notch.

### PI3K stabilizes NICD to promote Notch signaling

We next examined whether PI3K regulated the proteolytic cleavage of the Notch protein. Time course analysis by western blot indicated that the Notch1 intracellular domain (N1ICD) was generated as early as 10 minutes after EDTA treatment and was blocked by DAPT (Fig 5D). In contrast, although increasing the concentration of LY294002 progressively quenched AKT phosphorylation at 10 minutes, it did not reduce the level of N1ICD until 1 hour after EDTA treatment. Nevertheless, 50 μM LY294002 still disrupted the induction of Hes1 by EDTA. These results suggested that PI3K did not influence the initial cleavage of the Notch1 receptor but rather affected the stability of N1ICD. To further corroborate this finding, we overexpressed a Human influenza hemagglutinin (HA)-tagged rat N1ICD in lens cells. After blocking protein synthesis using cycloheximide (Chx), LY294002 treatment lead to a sharp decline of N1ICD over the course of 6 hours, while the AKT level was maintained (Fig 5E). This effect was confirmed using another potent PI3K inhibitor, PX-866 (S2 Fig) [40]. Glycogen Synthase Kinase 3β (GSK3β), a protein previously reported to affect Notch signaling, can be phosphorylated by AKT to inhibit its activity [41, 42]. We found that LY294002 reduced phosphorylation of both AKT and GSK3β in the cultured lens cells. Although the addition of a potent and specific GSK3β inhibitor (GSK3βi) CHIR99021 had little effect on AKT and GSK3β phosphorylation, it partially reversed N1ICD instability caused by the LY294002 treatment (Fig 5G). These results support that GSK3β is a downstream mediator of PI3K in regulating Notch signaling.

**Fig 6:**
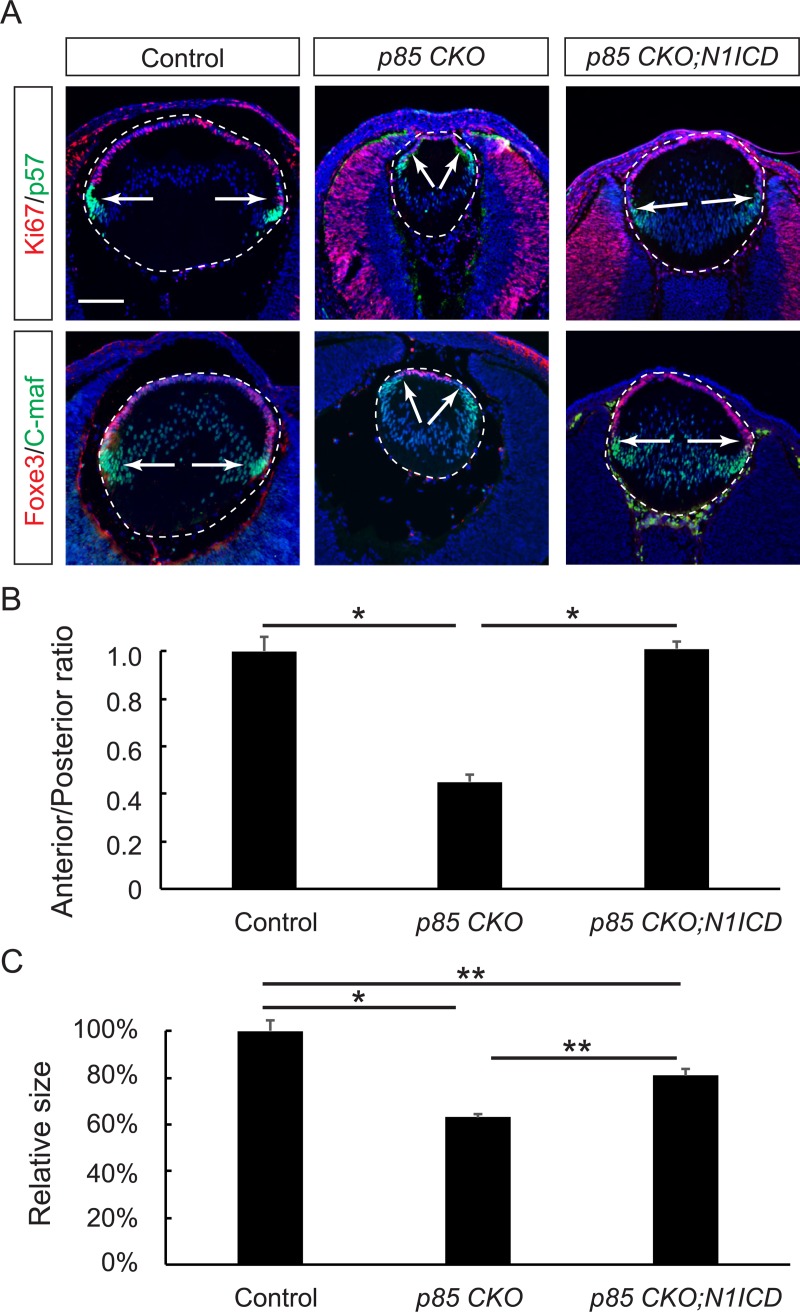
Overexpression of N1ICD compensated for the loss of *p85* in lens development. **(A)** As indicated by Ki67/Foxe3 and p57/C-maf staining (arrows), overexpression of NICD in *p85 CKO* reverted the transitional zone to the equatorial region (arrows). **(B)** Quantification of the anterior and posterior perimeter ratio. One-way ANOVA test, *P < 0.001, *n* = 9 embryos for control, *n* = 7 for *p85 CKO*, and *n* = 6 for *p85 CKO;N1ICD*. **(C)** Quantification of the lens size. One-way ANOVA test, *P < 0.001, **P < 0.05, *n* = 5 embryos for control, *n* = 3 for *p85 CKO*, and *n* = 4 for *p85 CKO;N1ICD*. The numerical data used in panels B and C are included in S1 Data. C-maf, MAF BZIP Transcription Factor; Foxe3, Forkhead Box E3; KO, knockout; NICD, Notch intracellular domain; N1ICD, Notch1 intracellular domain; *p85 CKO*, *Le-Cre;p85α*^*f/f*^*p85β*^*KO/KO*^; *p85 CKO;N1ICD*, *Le-Cre;p85α* ^*f/f*^*;p85β* ^*KO/KO*^*;Rosa*^*N1-IC*^.

If the role of PI3K in maintaining the lens progenitor cell pool is to promote the stability of N1ICD, we reasoned that overexpression of N1ICD may compensate for the loss of PI3K in lens development. To test this hypothesis, we crossed *p85 CKO* mutants with mouse carrying a *Rosa26* knock-in allele (*Rosa*^*N1-IC*^) that expressed N1ICD after the removal of the *loxP-STOP-loxP* cassette [43]. It was previously shown that overexpression of N1ICD in the lens resulted in hyperproliferation of the anterior lens epithelium at the expense of fiber-cell differentiation [36]. Likewise, *Le-Cre;p85α* ^*f/f*^*;p85β* ^*KO/KO*^*;Rosa*^*N1-IC*^ (*p85 CKO;N1ICD*) embryos displayed a significant expansion of the Ki67-positive lens epithelium compared to that of the *p85 CKO* mutants (Fig 6A). Marked by the boundary between Foxe3 and C-maf staining, the transitional zone was also reverted back to the equatorial region (Fig 6A and 6B). As a result, there was a significant increase in lens size in *p85 CKO;N1ICD* mutants (Fig 6C). This genetic evidence is in line with the model that PI3K stabilizes N1ICD to control lens differentiation.

### FGF–MAPK signaling crosstalk with PDGF–PI3K–Notch signaling in lens development

**Fig 7:**
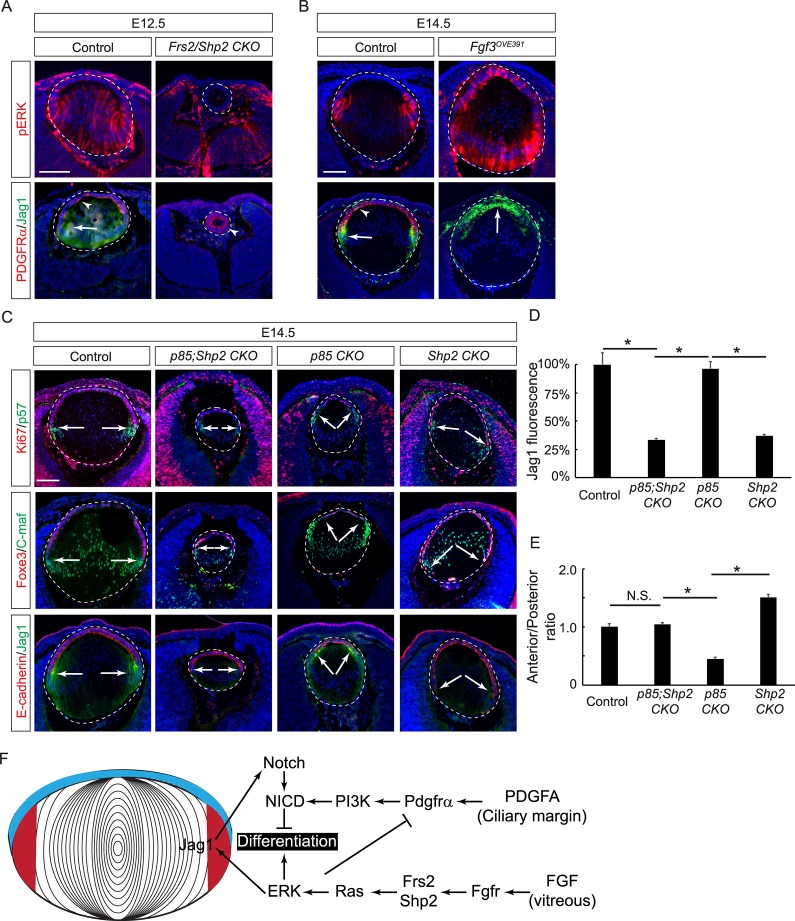
FGF–Shp2 signaling induces the expression of the Notch ligand while also antagonizing the PDGF–PI3K pathway. **(A)** Deletion of FGF-signaling mediators Frs2 and Shp2 abolished ERK phosphorylation and Jag1 expression, leaving the entire lens vesicle expressing PDGFRα. **(B)** Overexpression of *Fgf3* resulted in increasing ERK phosphorylation and anterior expansion of Jag1 expression at the expense of PDGFRα. Arrows and arrowheads point to Jag1 and PDGFRα staining, respectively. **(C)** In contrast to the *p85 CKO*, the *Shp2 CKO* lens had an extended lens epithelium arc. Deleting *Shp2* in the *p85 CKO* resulted in a smaller lens but a more balanced anterior to posterior ratio. **(D)** The relative fluorescent intensities of Jag1 at the transitional zones of the lenses. One-way ANOVA test, *P < 0.001, *n* = 3 embryos for each genotype. **(E)** Quantification of the anterior and posterior perimeter ratio. One-way ANOVA test, *P < 0.0001, *n* = 9 embryos for the control, *n* = 7 for *p85 CKO* and *p85;Shp2 CKO*, *n* = 11 for *Shp2 CKO*. **(F)** Model of PDGF, FGF, and Notch signaling crosstalk in lens development. FGF from the vitreous activates the Frs2/Shp2 mediated Ras–ERK pathway to promote cell differentiation, but it also induces Jag1 in the nascent lens fiber cells to activate Notch receptors in the adjacent lens epithelial cells. PDGFA from the ciliary margin activate PI3K signaling to stabilize the Notch effector NICD, which restrains FGF–MAPK-dependent cell differentiation at the transitional zone of the lens. The numerical data used in panels D and E are included in S1 Data. C-maf, MAF BZIP Transcription Factor; E, embryonic day; ERK, Extracellular Signal-Regulated Kinase; FGF, fibroblast growth factor; Foxe3, Forkhead Box E3; Frs2, Fibroblast Growth Factor Receptor Substrate 2; Jag1, Jagged 1; KO, knockout; MAPK, Mitogen-Activated Protein Kinase; NICD, Notch intracellular domain; PDGF, platelet-derived growth factor; PDGFRα, PDGF Receptor α; pERK, phospho-ERK; PI3K, Phosphoinositide 3-kinase; *p85 CKO*, *Le-Cre;p85α*^*f/f*^*;p85β*^*KO/KO*^; *p85;Shp2 CKO*, *Le-Cre;p85α*^*f/f*^*;p85β*^*KO/KO*^*;Shp2*^*f/f*^; Shp2, Src Homology Phosphatase 2; *Shp2 CKO*, *Le-Cre;Shp2*^*f/f*^; Src, Rous sarcoma oncogene.

The above results showed that the PDGF–PI3K signaling enhanced the Notch pathway to maintain the lens progenitor pool, which is opposite to the known function of FGF–ERK signaling in stimulating lens cell differentiation [5, 44]. This prompted us to examine the potential interactions between these two critical signaling pathways. We have previously shown that FGF receptors engage the docking protein Fibroblast Growth Receptor Substrate 2 (Frs2) and the tyrosine phosphatase Rous sarcoma oncogene (Src) Homology Phosphatase 2 (Shp2) with the purpose of inducing ERK signaling during lens development [45]. In support of this model, the genetic ablation of *Frs2* and *Shp2* abolished ERK phosphorylation in the *Le-Cre;Frs2* ^*f/f*^*;Shp2* ^*f/f*^ (*Frs2;Shp2 CKO*) lens at E12.5 (Fig 7A). In contrast to the control lens that expressed PDGFRα in the anterior and Jag1 in the posterior, *Frs2;Shp2 CKO* mutants contained a hollow lens with only PDGFRα expression (Fig 7A, arrow and arrowheads). In a complementary approach, we also examined a transgenic mouse line (*Fgf3*^*OVE391*^) that overexpressed *Fgf3* under control of an αA-crystallin promoter [46, 47]. As expected, pERK staining expanded from the transitional zone to the entire *Fgf3*^*OVE391*^ lens, coinciding with ectopic induction of Jag1 and loss of PDGFRα expression in the anterior lens epithelium (Fig 7B, arrow and arrowheads). These results demonstrated that FGF signaling regulates the Notch pathway by inducing the expression of its ligand as well as inhibiting PDGF signaling by suppressing its receptor.

We next investigated the signaling crosstalk at the level of MAPK and PI3K. To avoid the early lens defect in *Frs2;Shp2 CKO* mutants, we ablated only *Shp2* in the lens, which we previously showed to attenuate MAPK signaling, reduce cell proliferation, and increase apoptosis [48]. Interestingly, we observed a modest but consistent reduction in Jag1 expression in the *Le-Cre;Shp2* ^*f/f*^ (*Shp2 CKO*) mutant, indicating a dosage-dependent regulation of Jag1 by MAPK signaling (Fig 7C and 7D). Combined deletion of both *Shp2* and *p85* resulted in a smaller lens size than either single mutant, consistent with the notion that both MAPK and PI3K are required for cell survival and proliferation in the lens. Notably, the *Shp2 CKO* mutant displayed a posterior shift of the transitional zone, as opposed to the anterior shift observed in the *p85 CKO* lens. The transitional zone in the *Le-Cre; p85α* ^*f/f*^*;p85β* ^*KO/KO*^*;Shp2* ^*f/f*^ (*p85;Shp2 CKO*) lens, however, was restored to the equatorial region (Fig 7C and 7E). Taken together, these results revealed that MAPK and PI3K play the dual role of cooperating in promoting lens cell survival and proliferation while also antagonizing each other in the regulation of cell differentiation.

## Discussion

PDGF and FGF receptors are both RTKs that share a similar network of signaling pathways. Previous studies using cell-culture models have identified the potential downstream effectors of PDGF signaling as PI3K [20], Src [49], Phospholipase C γ (PLCγ) [50], and MAPK cascades [51], many of which are also activated by FGF signaling [52]. In this study, we showed that under physiological conditions, FGFRs and PDGFRs within the lens were biased toward different downstream effectors: FGFRs mainly activated Ras–MAPK, while PDGFRs mainly activated PI3K–AKT. This arrangement sets up both positive and negative interactions between these two RTK signaling pathways (Fig 7F). On the cooperative side, the FGF–Ras–MAPK pathway induces the Notch ligand Jag1, while the PDGF–PI3K–AKT one stabilizes the Notch effector NICD. This leads to the activation of Notch signaling with the purpose of dampening lens cell differentiation. On the antagonistic side, MAPK restricts PI3K activity by inhibiting PDGFRα expression while also directly promoting lens cell differentiation. By biasing progenitor cells toward distinct cell fates, the FGF–MAPK and PDGF–PI3K pathways enable the lens to achieve the optimum balance of expansion versus differentiation during development.

Both PDGF and PI3K signaling have been previously investigated for their roles in lens development. Genetic perturbation of PI3K signaling by deleting the catalytic subunit *p110α* or the negative regulator *Pten* have revealed its role in lens cell growth and survival [28, 53]. In vitro studies, however, have shown that the PDGF–PI3K signaling pathway may also promote lens-fiber–cell differentiation [12, 21, 27, 54, 55]. It was therefore a surprise to find that the complete ablation of PDGFRα and PI3K signaling in vivo resulted in premature differentiation and depletion of the lens progenitor cells. This discrepancy is likely due to the different experimental systems used. The previous in vitro studies were 2D models that utilized lens cell lines and explant cultures that expose all the cells to a uniform environment. In contrast, our in vivo study showed that the lens epithelial cells experienced not only the prodifferentiation FGF signal from the vitreous humor but also the antidifferentiation Notch signal from the fiber cells. In this 3D environment, PDGF–PI3K signaling was restricted to the transitional zone of the lens because of the convergent expressions of the ligand (PDGFA), receptor (PDGFRα), and regulator (p85). This confined the thrust of PDGF–PI3K signaling to the augmentation of Notch signaling in maintaining the lens progenitor cell population. This highlights the importance of the precise environmental context with regards to understanding the different functions of cell signaling.

Notch signaling is an evolutionarily conserved pathway controlling the proliferation and differentiation of progenitor cells [56, 57]. It is the underlying mechanism for lateral inhibition, which enables equipotent cells to take on divergent cell fates. In this model, the newly differentiated cells express high levels of the ligand, Jagged or Delta, that activates the Notch receptor in the neighboring progenitor cells. This leads to proteolytic cleavages of Notch adjacent to the transmembrane domain by Tumor Necrosis Factor, Alpha, Converting Enzyme (TACE) and at its transmembrane domain by γ-secretase. The resulting cytoplasmic peptide of Notch (NICD) translocates to the nucleus, where it binds the transcriptional factors Recombination Signal Binding Protein For Immunoglobulin Kappa J Region (Rbpj) and recruits Mastermind to activate downstream genes. These target genes, including the family of BHLH transcription factors, Hes and Hes Related Family BHLH Transcription Factor With YRPW Motif (Hey), act in progenitor cells to prevent differentiation and maintain the cells’ proliferative abilities [56, 58]. PI3K has been previously implicated in Notch signaling. In mouse embryonic fibroblast (MEF) and a variety of human cancer cell lines, PI3K has been shown to induce Jag1 expression via the Mammalian Target Of Rapamycin Kinase (mTOR) pathway to positively regulate Notch signaling [59]. In the *Drosophila* external sensory organ and Human embryonic kidney 293 (HEK293) cells, however, the mTOR regulator tuberous sclerosis complex (TSC) was reported to activate Notch in a target of rapamycin complex 1 (TORC1)-independent fashion [60]. Another potential mediator of PI3K regulation of Notch is GSK3β, which can be inactivated by AKT phosphorylation. GSK3β has been shown to bind and phosphorylate NICD in vitro [42, 61]. Although *GSK3β*-null MEF cells appeared to exhibit a reduced level of Notch signaling, it was later reported that the inhibition of GSK3β by PI3K–AKT promoted Notch signaling in a variety of different cell types that included Chinese hamster ovary (CHO) cells, T cells, and hippocampal neurons [41, 61]. Consistent with this, the genetic ablation of *GSK3α* and *GSK3β* in the developing cortex resulted in an elevated level of NICD and Hes1 [62]. In our study, we did not observe any decrease in Jag1 expression in the *p85 CKO* mutant lens. Instead, the inhibition of PI3K accelerated the degradation of NICD in lens cells and could be partially rescued through the blocking of GSK3β activity. These results suggested that the stabilization of NICD through the inhibition of GSK3β activity is an important mechanism for PI3K to potentiate Notch signaling in lens development.

A major goal in studying multicellular organisms is to understand the cellular algorithm by which overlapping signals exert distinct biological outcomes. Using lens development as a model, we have revealed in this study that FGF and PDGF receptors both cooperate and antagonize each other in a tightly regulated fashion. What accounts for the functional differences between these two similar RTKs? First, despite their propensities to induce the same set of downstream signaling cascades, RTKs differ in their mode of engagement with these pathways. Whereas PDGFR directly binds the p85 subunit of PI3K to activate PI3K–AKT signaling, the main signaling output of FGFR is channeled through the adaptor protein Frs2, which recruits Shp2, Growth Factor Receptor Bound Protein 2 (Grb2), and v-crk avian sarcoma virus CT10 oncogene homolog (Crk) proteins to activate the Ras–MAPK signaling cascade [47, 63]. Although Grb2 has been reported to interact with GRB2-Associated Binding Protein 1 (Gab1) to activate PI3K, we previously showed that the genetic ablation of Gab1 and its homologue Gab2 failed to disrupt FGF signaling in both MEF cells and the developing lens [45, 64]. Thus, the intrinsic disparities in the signaling networks bias PDGFR toward PI3K and FGFR toward MAPK, which has been documented in a variety of cell types and demonstrated in genetic studies [20, 22–24]. The differential responses to FGF and PDGF were also evident in early studies using lens epithelial explants that found that compared to FGF, PDGFA led to more rapid increase in AKT phosphorylation but less sustained ERK phosphorylation [12, 65]. Such signaling biases certainly do not equate to ERK activation exclusively by FGF or PI3K activation solely by PDGF, but they help to explain the distinct physiological functions of these two growth factors. Second, under physiological conditions, the ligands and receptors of RTK signaling differ in their affinities and stoichiometry, which can lead to quantitative differences in the duration and amplitude of intracellular pathways as well as qualitative differences in the specific substrates being recruited [28, 66]. Notably, such signaling specificity can also be overridden in nonphysiological conditions, which may explain why the transgenic overexpression of *Pdgfa* could promote lens cell differentiation and the constitutive activation of PI3K after *Pten* deletion could ameliorate some of the *Fgfr2* lens KO phenotype characteristics [9, 28]. The third reason why PDGF and FGF signaling leads to different cell fate decisions in lens development has to do with their involvement in Notch signaling. We have shown that while FGF–MAPK signaling induces Notch ligand expression, the PDGF–PI3K pathway stabilizes the Notch effector NICD. Because of the lateral inhibition effect of Notch signaling, cell differentiation is permitted in the Notch-ligand–expressing cells but restrained in the Notch effector cells. This naturally segregates the biological functions of FGF–MAPK and PDGF–PI3K signaling into pro- and antidifferentiation outcomes in lens development. Thus, the cooperative and inhibitory interactions among FGF, PDGF, and Notch pathways can be considered as “and” and “or” gates of signaling circuitry, which are used to generate intricate programs that achieve the exquisite balance of growth and differentiation in a complex multicellular organism.

## Materials and methods

### Ethics statement

The animal experiments were approved by Columbia University Institutional Animal Care and Use Committee (protocol number: AAAR0429). The mice were killed in humane fashion via CO_2_ asphyxiation and cervical dislocation according to the American Veterinary Medical Association (AVMA) Guidelines.

### Mice

Mice carrying *Frs2α*^*flox*^ and *Shp2*^*flox*^ alleles were bred and genotyped as described [67, 68]. We obtained *Fgf3*^*OVE391*^ from Dr. Michael Robinson (Miami University, Oxford, OH, USA), *Le-Cre* from Drs. Ruth Ashery-Padan (Tel Aviv University, Tel Aviv, Israel) and Richard Lang (Children's Hospital Research Foundation, Cincinnati, OH, USA), *p85α*^*flox*^ and *p85β*^*KO*^ from Dr. Lewis Cantley (Weill Cornell Medicine, New York, NY, USA), and *Pdgfrα*^*ΔPI3K*^ from Dr. Philipo Soriano (Mount Sinai School of Medicine, New York, NY, USA) [16, 20, 25, 26, 46, 69]. *Pdgfrα*^*flox*^ (stock no.: 006492), *Pdgfrα*^*GFP*^ (stock no.: 007669), and *Pten*^*flox*^ mice (stock no.: 006440) and *Rosa-N1-ICD*^*flox/+*^ (stock no.: 008159) and *Bax*^*flox/flox*^*;Bak*^*KO/KO*^ (stock no.: 006329) mice were obtained from Jackson Laboratory (Bar Harbor, ME, USA) [31, 32, 43, 70–72]. In all conditional KO experiments, mice were maintained on a mixed genetic background, and *Le-Cre*–only or *Le-Cre* and heterozygous flox mice were used as controls. Mouse maintenance and experimentation were performed according to protocols approved by Columbia University Institutional Animal Care and Use Committee.

### Histology, immunohistochemistry, and RNA in situ hybridization

Mouse embryos were fixed with 4% paraformaldehyde (PFA) in PBS overnight and paraffin- or cryo-embedded. The paraffin sections (10 μm) were rehydrated and stained with hematoxylin–eosin (HE) for histological analysis or TUNEL and BrdU staining as previously described [73, 74]. RNA in situ hybridization and immunostaining were performed on at least three cryosections for each embryo (10μm) [75]. The in situ probes used were *Jag1* (from Doris Wu, National Institute on Deafness and Other Communication Disorders) and *Pdgfrα* (Marc Mercola, Harvard Medical School). The following primary antibodies were used: anti-C-maf (sc-7866), ant-Foxe3 (sc-377465), and anti-Jag1 (sc-6011) (all from Santa Cruz Biotechnology, Dallas, TX, USA); anti-p85 (#4257), pAKT (D9E), and anti-pERK1/2 (#4370) (all from Cell Signaling Technology, Danvers, MA, USA); anti-P57 (ab75974, from Abcam, Cambridge, UK); anti-E-cadherin (U3254, from MilliporeSigma, St. Louis, MO, USA); and anti-Ki67 (#550609) and anti-Pdgfrα (#558774) (both from BD Pharmingen, BD Biosciences, San Jose, CA, USA). The antibody against Hes1 was a gift from Dr. Ryoichiro Kageyama (Kyoto University, Kyoto, Japan). Antibodies against α-, β-, and γ-crystallins were kindly provided by Dr. Sam Zigler (National Eye Institute). For pERK and pAKT staining, the signal was amplified using a Tyramide Signal Amplification kit (TSA Plus System, PerkinElmer Life Sciences, Waltham, MA, USA). To analyze the fluorescent intensity, the average pixel intensity in the lens transitional zone was obtained by measuring the grayscale value using ImageJ (NIH), and each data point was calculated by taking the average from three separate images of the same lens. The lens anterior and posterior arcs and areas were measured using ImageJ and analyzed by one-way ANOVA analysis. If not otherwise stated, at least three embryos were analyzed for each genotype, and they presented a consistent phenotype.

### Generation of immortalized lens cells

Ten newly born mouse pups were dissected in cold PBS, and the lenses were rolled over on an autoclaved filter paper before being transferred into 0.5 ml of cold PBS. These lenses were treated with 0.125% trypsin in EMEM (Eagle’s MEM), minced using 22-gauge needles, and incubated for 5 minutes at 37°C. The EMEM plus 4 mM L-Glutamine with 10% fetal bovine serum (FBS) was added to stop trypsin activity. After centrifuge, the lens cells and tissue pieces were plated in a 35-mm petri dish. Because of lack of proliferative ability, lens fiber cells eventually died out after 4 days of culture. After 3 days of exposure to SV40 T-antigen retroviral particles collected from a Ψ2 cell supernatant, immortalized lens cells were maintained in DMEM with 10% FBS [19]. The lens cells were starved prior to stimulation by FGF2 and PDGFA.

### Cell protein extraction and western blot

Cells in a 24-well plate were lysed with 80 μl/well RIPA buffer (50 mM Tris-HCl [pH 8.0], 150 mM NaCl, 1% NP40, 0.5% sodium deoxycholate, 0.1% SDS) supplemented with 1× Halt protease inhibitor cocktail (ThermoFisher, Waltham, MA, USA). Cell lysates were denatured by boiling with Laemmli SDS sample buffer for 5 minutes before protein separation on 8%–10% SDS polyacrylamide gels. After protein transfer, the Immobilon-FL PVDF membrane (Millipore) was blocked with Odyssey TBS blocking buffer (LI-COR Biosciences, Lincoln, NE, USA) at room temperature for 1 hour. Primary antibodies were diluted in the same blocking buffer with 0.1% Tween-20 and detected by corresponding secondary antibodies conjugated with IRDye 800CW or 680RD (LI-COR Biosciences). Proteins were visualized by an infrared-based Odyssey SA scanner (LI-COR Biosciences). The signal intensity was quantified using the Odyssey software. The antibodies used for western blot were mouse anti-pERK1/2 (sc-7383), anti-HA (sc-805), and anti-Hes1 (sc-25392) (all from Santa Cruz Biotechnology) and mouse anti-AKT (#4060), anti-ERK1/2 (#4695) anti-Notch1-ICD (#4147), rabbit anti-pAKT (#4060), and anti-phospho-GSK3β (pGSK3β) (#9336) (all from Cell Signaling Technology).

### Notch activation and degradation assay

For the Notch activation assay, 3.5 × 10^3^ immortalized lens cells were plated in each well of a 24-well tissue culture plate two days before the experiment began. Cells were first pretreated with the PI3K inhibitor LY294002 (10 μM or 50 μM) or the γ-secretase inhibitor DAPT (20 ng/μl) for 1 hour before treatment with 1 μM EDTA in Hanks’ balanced salt solution for 15 minutes to activate Notch. Cells were then incubated in DMEM with and without LY294002 or DAPT for another 10 minutes or 1 hour before protein or RNA extraction and analysis by western blots or qPCR.

To measure N1ICD degradation, 3.5 × 10^3^ immortalized lens cells were transfected with 0.5 μg plasmid pCCL-Notch1IC (a gift from Dr. Jan Kitajewski, University of Illinois Cancer Center, IL) using Lipofectamine 3000 (Invitrogen, Carlsbad, CA, USA) following the manufacturer’s protocol. This plasmid contains a cDNA encoding N1ICD (rat: NM_001105721.1) with a C-terminal HA tag. 24 hours after transfection, cells were treated with 50 μM LY294002, 1 μM PX-866, or 5 μM CHIR99021 in the presence of protein synthesis inhibitor Chx (300 ng/μl). Cell lysates were collected before or after 1.5 hours, 3 hours, 4.5 hours, or 6 hours of treatment. The levels of N1ICD were quantified by western blot analysis probed with an anti-HA-tag antibody.

### Total RNA extraction and qPCR

Cells in a 24-well plate were lysed with 500 μl/well TRIzol Reagent (ThermoFisher), and total RNA was extracted according to the manufacturer’s protocol. cDNAs were synthesized using the High-Capacity cDNA Reverse Transcription Kit (ThermoFisher). Triplicate PCR reactions were prepared with SYBR Green PCR master mix (Applied Biosystems, Foster City, CA, USA) and carried out in a StepOnePlus real-time PCR system (Applied Biosystems). The primers used for *Hes1* are 5′-TCAACACGACACCGGACAAAC and 5′-ATGCCGGGAGCTATCTTTCTT. 18S rRNA (5′-GTAACCCGTTGAACCCCATT, 5′-CCATCCAATCGGTAGTAGCG) was amplified as the internal control. The C_T_ value of each reaction was used to calculate the relative concentration of the target RNAs.

## Supporting information

S1 Fig Characterization of immortalized lens epithelial cells. Some of the immortalized lens cells expressed high levels of Pax6 and α-crystallin, whereas others expressed β- and γ-crystallin, suggesting a mixture of progenitor and more differentiated cell types. Pax6, Paired Box 6.
(EPS)

S2 Fig PI3K inhibitor PX-866 promotes degradation of N1ICD. 1 μM PX-866 treatment abolished AKT phosphorylation and promoted degradation of N1ICD. Quantification of the HA–N1ICD level normalized with AKT expression after two independent experiments. The numerical data are included in S1 Data. AKT, Protein kinase B; HA, Human influenza hemagglutinin; N1ICD, Notch1 intracellular domain; PI3K, Phosphoinositide 3-kinase.
(EPS)

S1 Data Data underlying this study. (XLSX)

## Abbreviations

AKT: Protein kinase B
AVMA: American Veterinary Medical Association
Bak: BCL2 Antagonist/Killer
Bax: BCL2-Associated X
BCL-2: B-cell lymphoma 2
BHLH: basic helix–loop–helix
BrdU: Bromodeoxyuridine
CHO: Chinese hamster ovary
Chx: cycloheximide
C-maf: MAF BZIP Transcription Factor
Crk: v-crk avian sarcoma virus CT10 oncogene homolog
DAPT: N-[N-(3,5-Difluorophenacetyl)-L-alanyl]-S-phenylglycine t-butyl ester
E: embryonic day
EMEM: Eagle’s MEM
ERK: Extracellular Signal-Regulated Kinase
FBS: fetal bovine serum
FGF: fibroblast growth factor
FGFR: FGF receptor
Foxe3: Forkhead Box E3
Frs2: Fibroblast Growth Factor Receptor Substrate 2
Frs2: *Shp2 CKO*, *Le-Cre;Frs2*^*f/f*^*;Shp2*^*f/f*^
Gab1: GRB2 Associated Binding Protein 1
GFP: green fluorescent protein
Grb2: Growth Factor Receptor Bound Protein 2
GSK3β: Glycogen Synthase Kinase 3β
GSK3βi: GSK3β inhibitor
HA: Human influenza hemagglutinin
HE: hematoxylin–eosin
HEK293: Human embryonic kidney 293
Hes1: Hes Family BHLH Transcription Factor 1
Hey: Hes Related Family BHLH Transcription Factor With YRPW Motif
Jag1: Jagged 1
KO: knockout
MAPK: Mitogen-Activated Protein Kinase
MEF: mouse embryonic fibroblast
mTOR: Mammalian Target Of Rapamycin Kinase
NICD: Notch intracellular domain
N.S.: not significant
N1ICD: Notch1 intracellular domain
pAKT: phospho-AKT
Pax6: Paired Box 6
PDGF: platelet-derived growth factor
PDGFRα: PDGF Receptor α
pERK: phospho-ERK
PFA: paraformaldehyde
pGSK3β: phospho-GSK3β
PI3K: Phosphoinositide 3-kinase
PI-3: 4,5-P, Phosphatidylinositol (3,4,5)-trisphosphate
PI-4: 5-P, Phosphatidylinositol 4,5-bisphosphate
PLCγ: Phospholipase C γ
Pten: phosphatase and tensin homolog
p85 CKO: *Le-Cre;p85α*^*f/f*^*;p85β*^*KO/KO*^
p85 CKO: *N1ICD*, *Le-Cre;p85α*^*f/f*^*;p85β*^*KO/KO*^*;Rosa*^*N1-IC*^
p85: *Bax/Bak CKO*, *Le-Cre;p85α*^*f/f*^*;p85β*^*KO/KO*^*;Bax*^*flox/flox*^*;Bak*^*KO/KO*^
p85: *Pten CKO*, *Le-Cre;p85α*^*f/f*^*;p85β*^*KO/KO*^*;Pten*^*f/f*^
p85: *Shp2 CKO*, *Le-Cre;p85α*^*f/f*^*;p85β*^*KO/KO*^*;Shp2*^*f/f*^
Rbpj: Recombination Signal Binding Protein For Immunoglobulin Kappa J Region
RTK: Receptor Tyrosine Kinase
RT-PCR: Reverse transcription-polymerase chain reaction
Shp2: Src Homology Phosphatase 2
Shp2 CKO: *Le-Cre;Shp2*^*f/f*^
Src: Rous sarcoma oncogene
TACE: Tumor Necrosis Factor, Alpha, Converting Enzyme
TORC1: target of rapamycin complex 1
TSC: tuberous sclerosis complex
